# Immunomodulating Activity of *Nymphaea rubra* Roxb. Extracts: Activation of Rat Dendritic Cells and Improvement of the T_H_1 Immune Response

**DOI:** 10.3390/ijms130910722

**Published:** 2012-08-24

**Authors:** Jai-Hong Cheng, Shau-Yu Lee, Yi-Yang Lien, Meng-Shiou Lee, Shyang-Chwen Sheu

**Affiliations:** 1 The Department of Nursing, Shu Zen College of Medicine and Management, Kaohsiung 821, Taiwan; E-Mail: cjaiho@yahoo.com.tw; 2 Department of Food Science, National Pingtung University of Science and Technology, No. 1, Shuehfu Rd., Neipu, Pingtung 91201, Taiwan; E-Mail: leemengshiou@yahoo.com.tw; 3 Department of Veterinary Medicine, National Pingtung University of Science and Technology, No. 1, Shuehfu Rd., Neipu, Pingtung 91201, Taiwan; E-Mail: yylien@mail.npust.edu.tw; 4 Department of Chinese Pharmaceutical Science and Chinese Medicine Resources, China Medical University, Taichung 40402, Taiwan

**Keywords:** *Nymphaea rubra* Roxb., pullulanase, polysaccharides, dendritic cells, T_H_1 immune response, functional foods

## Abstract

Polysaccharides play a key role in enhancing immune function and facilitating cellular communication. Here, we purified *Nymphaea rubra* Roxb. polysaccharides (NR-PS) by treating them with pullulanase. They were then cultured with immature dendritic cells (DCs) derived from rat bone marrow hematopoietic cells (BMHCs). After treatment with bioactive NR-PS with a degree of polymerization (DP) value of 359.8, we found that the DCs underwent morphological changes indicative of activation. CD80/86 (87.16% ± 8.49%) and MHC class II (52.01% ± 10.11%) expression levels were significantly up-regulated by this treatment compared to the controls (65.45% ± 0.97% and 34.87% ± 1.96%). In parallel, endocytosis was also reduced (167.94% ± 60.59%) after treatment with 25 μg/mL of NR-PS as measured by the medium fluorescence intensity compared to the control (261.67% ± 47.26%). Furthermore, the DCs after treatment with 25 μg/mL NR-PS showed increased IL-12 (102.09 ± 10.16 to 258.78 ± 25.26 pg/mL) and IFN-γ (11.76 ± 0.11 to 15.51 ± 1.66 pg/mL) secretion together with reduced IL-10 secretion (30.75 ± 3.35 to 15.37 ± 2.35 pg/mL), which indicates a T_H_1 immune response. In conclusion, NR-PS exhibits stimulatory effects on rat DCs and promotes the secretion of T_H_1 cytokines. Taken together, our studies are the first to show that NR-PS is an immunomodulator affecting the maturation and functioning of DCs.

## 1. Introduction

The Nymphaeaceae include a wide range of flowering plants. They are also called water lilies and are distributed in tropical areas around the world, living on the banks of lakes and rivers [1,2]. The plants have a broad range of flower colors, including white, yellow, red and blue [3]. They are a perennial rhizomatous herb made up of six genera, namely Nymphaea, Nuphar, Barclaya, Victoria, Euryale and Ondinea, which include seventy species. Nymphaea is the major and most widely distributed genus and consists of 35 species of water lilies [2]. Many species of Nymphaea in Nepal, India and China are thought to acts as functional drug plants. Previous studies have reported that extracts of various organs from Nymphaea can be used as medicinal plant material. These include extracts of the rhizomes and flowers, which have anti-diabetic and anti-inflammatory effects [4–7], extracts of the rhizomes and seeds, which have the immunomodulatory activity [8], extracts of the stalks, which have an anti-pyretic effect [9], extracts of the leaves, flowers and stamens, which have been shown to have anti-oxidant effects [2,10,11], and extracts of seeds, which have been reported to have hepatoprotective and free radical scavenging effects [2,7,12,13].

Polysaccharides are polymers that consist of long carbohydrate molecules and are composed of monomer units linked together through glycosidic bonds [14]. There are a number of different types of polysaccharides in nature, such as storage polysaccharides (starches and glycogen) [15,16], structural polysaccharides (arabinoxylans, cellulose, chitin and pectins) [15,16], acidic polysaccharides [17] and bacterial polysaccharides (bacterial capsular polysaccharides) [1,14]. The polysaccharides extracted from higher plants, yeasts, fungi, bacteria and algae have been studied and are known to have a significant effect on the immune system. Polysaccharides have long been believed to have many different biological properties and certain polymers have recently been revealed to act as effective immunomodulating agents. However, while quite a few polysaccharide immunomodulators have been identified, most of these studies seem to be relatively unreliable and only a few polysaccharides have been examined in detail. Specifically, one example that has been studied in detail is the β-glucans and this has included the structure, functions and mechanisms of action of this type of polysaccharide.

It is thought that immunomodulating agents show antitumor activity by activating macrophages and natural killer cells via surface receptor binding [18,19]. Treatment with β-glucan polymers has been shown to result in an increased numbers of neutrophils and eosinophils as well as to cause an alteration in macrophage morphology. A previous report has show that mouse spleen cell proliferation is improved and that Sarcoma 180 tumor cell line growth is suppressed after treatment with polysaccharide from *Flammulina velutipes*, namely β-(1–3)-d-glucan [20]. Polysaccharides have also been shown to promote the innate immune system by interacting with dendritic cells (DCs), which are antigen-presenting cells that provide an important connection between innate and adaptive immunity [21]. Polysaccharides stimulate the maturation of DCs, allowing them to express MHC class II molecules as well as co-stimulatory molecules such as CD80/86 and CD40, all of which are essential for effective antigen presentation [22,23]. It is also known that several cytokines can be released by DCs, including IFN-γ, IL-12, and IL-4, and that these guide the development of T-cells down the path of cell-mediated or humoral immunity depending on the type of antigen captured and the maturation process [24–26].

*Nymphaea rubra* Roxb., a member of the *Nymphaea* genus, is grown mainly in India, where it is known as red water lily. The present study is the first to report polysaccharides from the carpel of the flowers of this plant as having immunomodulating activity. In addition, the immune cell-mediated functions of these polysaccharides are also explored. The present findings should help with the development and formulation of beneficial supplementary foods that ought to improve health via an improved innate immune system.

## 2. Results and Discussion

### 2.1. Extraction and Analysis of *Nymphaea rubra* Roxb. Composition

**Figure 1:**
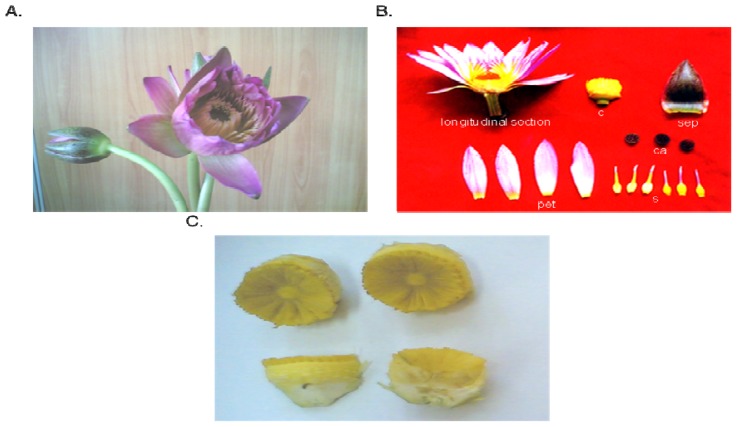
(**A**) The flower of *Nymphaea rubra* Roxb.; (**B**) Schematic of a longitudinal section through a quartered portion of the flower, (sep) sepals, (pet) petals, (s) stamen, (ca) carpellary appendage, (c) carpel; (**C**) The carpel of *Nymphaea rubra* Roxb.

The carpels of *Nymphaea rubra* Roxb. were collected (Figure 1C) and ground to give a crude extract. We then prepared both crude and hydrolyzed *Nymphaea rubra* Roxb. extracts and analyzed their composition. As shown in Table 1, the yield of crude extraction was 1.43% (wet weight). As previously reported [27,28], β-1,3-d-glucan has important biological functions and therefore pullulanase was added to the crude extract to hydrolyze any α-1,6 linkages present and to increase the solubility of the crude extract without breaking the β-1,3-glycosidic linkages. The yield of the hydrolyzed sample was 42.00% (dry weight) relative to the crude extract. The total sugar content and reducing sugar content of the hydrolyzed extract were 51.3% and 2.45%, respectively. The total protein content of the crude and hydrolyzed extracts were 7.84% and 5.28% respectively. The DP value of the hydrolyzed extract was found to be at 359.8 kDa by Glass Capillary Viscometer with a degree of branching (DB) value of 0.36. Bohn and BeMiller reported that the most active polysaccharide polymers have a high molecular weight (up to 100 kDa) and a DB between 0.2 and 0.5 [29]. Ohno *et al.* reported that the polysaccharide fractions prepared from cultured *A. blazei* containing a highly branched 1,3-β-glucan segment are the most active and have the highest antitumor activity and immunomodulary effects [30]. The *Nymphaea rubra* Roxb. polysaccharides (NR-PS) preparation used in this study had a DB of 0.36 and was within the bioactive range of polysaccharide polymers.

### 2.2. Morphological Changes and Activation of BMHC-Derived Immature Dendritic Cells (BHMC-imDCs) after NR-PS Treatment

**Figure 2:**
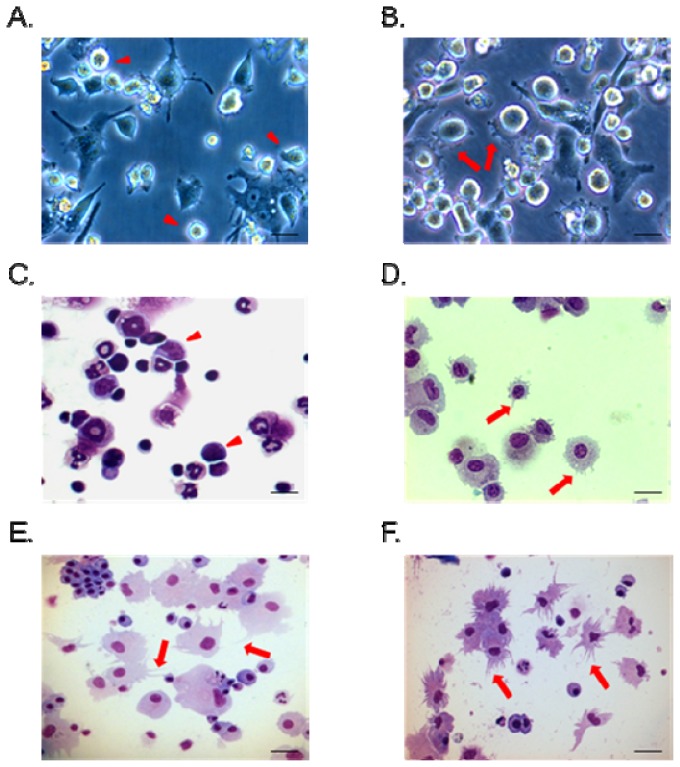
Morphology of dendritic cells after various treatments. (**A**) Bone marrow hematopoietic cells-imdendritic cells (BMHC-imDC) were grown from rat bone marrow in completed RPMI media 1640 containing 20 ng/mL recombinant rat GM-CSF and 10 ng/mL IL-4; (**B**) BMHC-imDC treated with 50 μg/mL NR-PS on day seven (200×); (**C**) A photomicrogaph of rat bone marrow hematopoietic cells (BMHC) isolated from four to seeks week old SD rats after staining with Liu’s-stain (200×); (**D**) BMHC-imDCs were generated from BMHC by treatment with 20 ng/mL GM-CSF and 10 ng/mL IL-4 for seven days. Note the presence of the short dendritic processes (arrow) associated with the plasma membrane (Liu’s staining, 200×); (**E**) Lipopolysaccharide(1 μg/mL) was used to stimulate the maturation of BMHC-imDC into BMHC-mDC. Note the presence of the long dendritic processes (arrow) associated with the plasma membrane (Liu’s staining, 200×); (**F**) Bone marrow hematopoietic cell-derived immature dendritic cells (BMHC-imDCs) treated with 50 μg/mL NR-PS for 48hr (Liu’s stain, 200×).

Bone marrow-derived DC (BMDC) maturation is attended by alteration of the cells’ morphological, phenotypic and functional properties [31,32]. According to Inaba *et al.*, it was found that treatment with GM-CSF and IL-4 was able to stimulate rat BMHCs to form dendritic cells and that similar results were obtained in mice [33]. So, we used GM-CSF and IL-4 to stimulate bone marrow hematopoietic cells (BMHCs) to differentiation and become BMHC-imDCs as Talmor *et al.* reported [34]. In order to measure the bioactivity of NR-PS using BMHC-imDCs, the morphological changes in BMHC-imDCs were monitored after NR-PS treatment. First, using live cell observation, BMHC-imDCs were obtained by treating BMHCs with rGM-CSF and rIL-4 for nine days without NS-PS and these cells were compared with others that had been treated with a test concentration of 50 μg/mL NS-PS at the immature dendritic cell stage on the seventh day and then cultured until the ninth day. The BMHC-imDCs were activated by NR-PS, and this was shown by the presence of dendritic protrusions on the cell surface (Figure 2A,B). Figure 2 shows the cells after staining with Liu’s-stain in order to observe any morphological changes. The BHMC-imDCs can be seen to be round with polymorphic nuclei and small protrusions on the cell surface in the absence of stimulation (Figure 2C,D). However, after treated with 50 μg/mL NR-PS (Figure 2F) or 1 μg/mL LPS (positive control) for 48 h (Figure 2E), the cells are now larger, and the nuclei have become even more polymorphic. Furthermore, the dendritic protrusions on the cell surfaces have become more pronounced and elongated [34]. These findings strongly suggest that NR-PS treatment promotes dendritic cell maturation.

### 2.3. CD11c, MHC Class II and CD80/86 Expression by Dendritic Cells after NR-PS Activation

**Figure 3:**
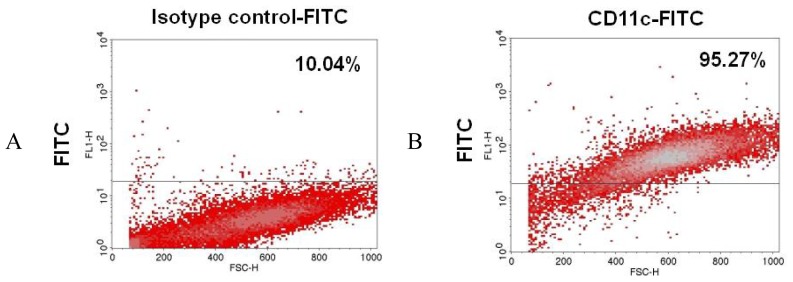
Analysis of the surface phenotypes of BMHC-imDC using fluorescence activated cell sorting (FACS) scan. On day nine, non-adherent cells were stained with (**A**) fluorescein isothiocyanate (FITC) conjugated isotype control; (**B**) conjugated anti-rat CD11c antibody.

Maturation of DCs is distinguished by a reduction in antigen-processing capacity together with an increase in cell surface expression of MHC class II molecules and the cell markers CD80, CD86, CD11c and CD40 [34,35]. The cell marker CD11c (LFA-1) was used to monitor the rat DCs [36,37]. After cytokine treatment, the BMHCs differentiated into BMHC-imDCs and these cells were shown to be 95.27% CD11c^+^ by fluorescence activated cell sorting (FACS) analysis (Figure 3). This process was used to set up a BMHC-imDCs assay system that measured the maturation of DCs after stimulation by NR-PS. In order to examine the activation of dendritic cells by NR-PS, the presence of the indicator molecules CD80 and CD86 as well as MHC class II expression were measured. In terms of T cell activation, MHC class II is functional during antigen presentation, while CD80 and CD86 (B7-1 and B7-2, respectively) are important co-stimulatory molecules. We used various concentrations of NR-PS (3.125, 6.25, 12.5, 25, 50 and 100 μg/mL) to treat the BMHC-imDCs for 48 h. The proportion of positive cells and the mean fluorescence intensity of the cells for CD80, CD/86 and MHCclass II were measured (Tables 2 and 3). When compared with the untreated control (65.45% ± 0.97% for positive cell ratio and 24.60% ± 3.19% for mean fluorescence intensity) and positive control treated with 1 μg/mL LPS (85.76% ± 3.06% for positive cell ratio and 49.00% ± 9.18% for mean fluorescence intensity), all NR-PS treatments showed an increase in the positive cell ratio (84.49% ± 10.86%, 84.80% ± 7.94%, 85.68% ± 18.66%, 87.16% ± 8.49%, 84.44% ± 6.35% and 84.56% ± 7.62%) (Table 2). There was a similar increase in mean fluorescence intensity to 47.23% ± 3.72%, 42.73% ± 4.20%, 45.69% ± 3.58%, 48.48% ± 4.26%, 38.03% ± 6.27% and 43.70% ± 7.61% (Table 2). Furthermore, similar results were also obtained for MHC class II expression. The untreated control was 34.87% ± 1.96% for the positive cell ratio and 46.55% ± 4.97% for mean fluorescence intensity, while the LPS positive control gave 73.20% ± 6.16% for positive cell ratio and 57.62% ± 1.87% for mean fluorescence intensity. On treatment with various concentrations of NR-PS, the positive cell ratio was increased to 45.06% ± 8.11%, 44.88% ± 5.11%, 49.18% ± 10.41%, 52.01% ± 10.11%, 49.27% ± 5.03% and 50.27% ± 5.24%, respectively (Table 3), while the mean fluorescence intensity was increased to 51.09% ± 4.73%, 51.94% ± 2.89%, 55.62% ± 8.21%, 60.74% ± 10.06%, 57.30% ± 4.76% and 57.70% ± 2.68%, respectively (Table 3). Thus treating DCs with NR-PS increases the positive cell ratio and the mean fluorescence intensity for all markers compared to the untreated control with the highest activation of the DCs cells being at 25 μg/mL NR-PS treatment (Tables 2 and 3). This confirms clearly that NR-PS is able to enhance the maturation of DCs.

### 2.4. The Endocytosis of Dendritic Cells Was Reduced after NR-PS Treatment

The expression of the mannose receptor is the determinant for the receptor-mediated endocytosis of dextran, and in mammals this receptor is expressed at high levels on immature DCs and is low or absent on mature DCs [38,39]. In the mammalian immune system, dendritic cells act as antigen presentation cells and function by endocytosing, processing and presenting antigens in order to activate the adaptive immune system [40,41]. As previously reported, immature DCs show higher levels of endocytosis and processing as mature DCs. In contrast, mature DCs show lower endocytosis, but have higher antigen-presenting activity [42]. Various concentrations of NR-PS were added in order to observe their effect on the level of endocytosis of BMHC-imDCs; this was done over 48 h using a FITC-dextran uptake assay (Table 4). The results showed that the mean fluorescence intensity was reduced to 167.94% after treatment with 25 μg/mL NR-PS compared to the control level of 261.67%. This reduction in endocytotic activity, as shown by the reduced dextran uptake, strongly suggests that NS-PS treatment leads to the activation of DCs.

### 2.5. NR-PS Treatment of Dendritic Cells Results in Increased IL-12/IFN-γ Secretion and Reduced IL-10 Secretion

Cytokines are secreted by DCs when they activate native CD4 T-cells, and these determine the developmental path of the CD4 T-cells. IL-12 and IFN-γ promote the development of CD4 T-cells into T_H_1 cells, while IL-10 favors development into T_H_2 cells. As shown in Table 5, both IL-12 and IFN-γ levels were elevated after treatment with various concentrations of NR-PS. When compared to the untreated group, the secretion of IL-12 and IFN-γ was significantly increased after treatment with 25 μg/mL NR-PS. IL-12 is an important cytokine involved in production of IFN-γ by T cells and NK cells and plays an important role in the differentiation of the T_H_1 cell population [18]. Moreover, the level of IL-10 secreted by the NP-PS treated cells decreased as the dose increased. When compared to the untreated control (30.75 ± 3.35 pg/mL), treatment with 25 μg/mL and 50 μg/mL NR-PS resulted in a reduction in IL-10 production to 15.37 ± 2.35 pg/mL and 14.83 ± 3.8 pg/mL (Table 5). These results were better than those reported previously by us for the functional food okra [27]. In the human immunosystem, DCs can modulate the T_H_1–T_H_2 balance according to their microbial interactions [43]. DCs accomplish this, at least in part, through differences in the production of IL-10 and IL-12, since IL-10 is concerned with initiating T_H_2 responses while IL-12 potently induces IFN-γ producing Th1 cells [44,45]. Taken together, the combined findings for DCs showing increased IL-12/IFN-γ and reduced IL-10 production indicates that NR-PS leads to a T_H_1 response.

## 3. Experimental Section

### 3.1. Rats

Four-week-old male CD (SD) IGS rats with initial weights between 75 g and 125 g were purchased from BioLasco (Taipei, Taiwan). In the experiments, the rats were fed a basal diet for more than a week with free access to water and feed to allow adjustment to their environment. The animal rooms were kept at 25 °C and 60% humidity with a 12 h light and dark cycle (8 am to 8 pm). All animal treatments were approved by the Animal Ethics Committee at the National Pingtung University of Science and Technology, Taiwan.

### 3.2. Preparation of Crude and Hydrolyzed *Nymphaea rubra* Roxb. Extract

*Nymphaea rubra* Roxb. (Figure 1) was purchased from the market. To produce the crude extract, the carpels of the flowers were cleaned, diced and immersed in 95 °C ddH_2_O for 4 h before centrifugation at 10,000× *g* for 10 min at 4 °C. Then 95% alcohol was used to precipitate the polysaccharides in the crude extract, and these were collected by centrifugation at 10,000× *g* for 10 min at 4 °C. An equal volume of acetone was added for decolorization before a second centrifugation at 10,000× *g* for 10 min at 4 °C. The residual acetone was allowed to evaporate in a hood, and the resulting crude extract was then lyophilized, ground and stored at −20 °C until use.

In order to prepare hydrolyzed *Nymphaea rubra* Roxb. extract, 5 mg of the crude extract was dissolved in 25 mL of 0.1 M sodium acetate buffer (pH 5.2), then 5 U of pullulanase (Sigma, USA) was added and the mixture incubated for 6 h at 45 °C. The reaction mixture was then cooled for 5 min on ice before being terminated with 20% trichloroacetic acid (TCA). The supernatant was collected by centrifugation at 10,000× *g* for 10 min at 4 °C. Ethanol to a final concentration of 80% was then added to allow precipitation, and the mixture was then incubated at 4 °C for 24 h. The precipitated polysaccharides were centrifuged at 10,000× *g* for 10 min at 4 °C, and then washed three times with 95% alcohol before being lyophilized, ground and stored at −20 °C until use.

### 3.3. Measurement of Sugar, Protein and Polymerization Levels of the *Nymphaea rubra* Roxb. Extract

Measurement of the total sugar content of the extract was carried out by a colorimetric method that has been previously described [46]. The total reducing sugar content was measured by the 3,5-dinitro-salicyclic acid method as previously described [47]. The absorbance of the sample solution was read at 540 nm. Glucose was used as a standard for both the total sugar content and reducing sugar content analyses. The protein concentration was determined by the Bradford method using a protein assay kit (Bio-Rad, USA) according the manufacturer’s instructions. Polymerization was measured by Glass Capillary Viscometer as previous reported [48].

### 3.4. Isolation of Rat BMHCs

We used the femur bones of sacrificed rats (between six and eight weeks old) to extract bone marrow using RPMI 1640 containing 5% FBS and 1% penicillin/streptomycin (P/S). The cells were collected and resuspended in RPMI 1640 complete medium (90% RPMI 1640 medium, 10% FBS, 1% penicillin-streptomycin, 2000 U GM-CSF, 10 U IL-4). Then 1 × 10^6^ cells/mL were seeded into a 75 cm^2^ culture flask and incubated at 37 °C with 5% CO_2_, and the medium was changed on the third and sixth day of culture.

Stimulation of the BMHC-imDCs involved treatment with NR-PS. On the sixth day of culture, the cells were collected by centrifugation, resuspended in RPMI 1640 complete medium, and seeded into 24-well flat bottom culture plates (1 × 10^6^ cells/well). The BMHC-imDCs were found to have differentiated after 24 h of incubation. On the seventh day the cells were treated with various concentrations of hydrolyzed NR-PS (0, 3.125, 6.25, 12.5, 25, 50, and 100 μg/mL) or 1 μg/mL lipopolysaccharide (LPS) (as a positive control) for 48 h at 37 °C with 5% CO_2_. Cell morphology, surface antigen presentation, cytokine secretion, and phagocytosis were then analyzed.

### 3.5. Observation of the Cell Morphology of the BMHC-imDCs

The morphology of the BMHCs was visualized using Liu’s stain, which comprises two staining solutions, namely Liu’s A stain (0.5 g/L methylene blue and 1.7 g/L eosin yellow in methyl alcohol) to visualize the cytoplasm, and Liu’s B stain (1.3 g/L azure and 1.4 g/L methylene blue) to visualize the nucleus. The cells were placed in a cytospin (Thermo Scientific, UK), centrifuged at 350× *g* for 10 min and stained with Liu’s reagent. The cell morphology was observed using a microscope.

### 3.6. Measurement of the BMHC-imDC Surface Markers by FACS Analysis

Cells (2 × 10^5^ cells/mL) were collected, washed with FACS buffer (1% BSA and 0.1% sodium acid in PBS) and resuspended in 100 μL of FACS buffer. Fluorescent monoclonal antibodies (2 μL) against CD11c, MHC class II and CD 80/86 surface antigens (eBioscience, USA) were added to the cells, and the mixture incubated in the dark for 30 min. Cells were then washed with FACS buffer and resuspended in 0.5 mL of 3.8% paraformaldehyde (in PBS), which was followed by analysis on a FACScan flow cytometer (Becton Dickinson, USA).

### 3.7. Assessing the Endocytotic Activity of BMHC-imDCs

In order to measure the endocytotic activity of the BMHC-imDCs, 4 μL of 1 mg/mL FITC-dextran (Sigma) was added to 96 μL of 1 × 10^6^ cells (in PBS buffer) that had been washed three times with PBS buffer. The cells were divided into two tubes. One tube was incubated at 4 °C for 1 h, and the other tube was incubated at 37 °C for 1 h. After the reaction, all cells were washed three times with cold PBS buffer (4 °C) and resuspended in 0.5 mL FACS buffer for analysis. The uptake of FITC-dextran was analyzed cytofluorimetrically using a FACScan flow cytometer (Becton Dickinson, USA).

### 3.8. Enzyme-Linked Immunosorbent Assay (ELISA) for Cytokine Detection

We used various concentrations of NR-PS (0, 3.125, 6.25, 12.5, 25, 50, and 100 μg/mL) to stimulate the BMHC-imDCs, and after nine days the supernatants were collected by centrifugation at 400× *g* for 8 min at 4 °C and were then stored at −80 °C. An IL-12 p40 ELISA kit was used to determine IL-12 expression according to the manufacturer’s instructions (Biosource, USA), while the amount of IFN-γ and IL-10 was determined using a cytokine assay kit (Bender, Austria) according to the manufacturers’ instructions.

### 3.9. Statistical Analysis

The data were analyzed using SAS software (SAS institute, USA) as previous described [27]. One-way analysis of variance (one-way ANOVA) and Duncan’s test were used to determine the statistical significance between groups. Differences were considered statistically significant at *p* ≤ 0.05.

## 4. Conclusion

This is the first study to examine the immunopotentiating effect of *Nymphaea rubra* Roxb. polysaccharides. This study demonstrates that NR-PS treatment promotes murine DC maturation as shown by the presence of morphological changes that are consistent with maturation, namely a reduction in endocytosis and an increase in the expression of surface molecules important for antigen presentation. Furthermore, NR-PS seems to induce a T_H_1 response. Our findings should help the development of new products that have health food applications, including functional foods that are able to immunomodulate. However, the production of *Nymphaea rubra* Roxb. extracts still needs to be improved in order to increase the production of β-glucan after pullulanase treatment. Further research is also needed to determine the best procedure for preparing functional foods containing *Nymphaea rubra* Roxb. So that they can be eaten directly and thus improve human immunity.

## Figures and Tables

**Table 1:** Composition of the crude extracts and hydrolysate from *Nymphaea rubra* Roxb.

Extract	Yield	Total sugar	Reducing sugar	Protein
Crude	1.43	55.86 ± 0.80 a	2.64± 0.34 a	7.84 ± 0.63 a
Hydrolyzed	42.00	51.30 ± 0.98 a	2.45 ± 0.92 a	5.28 ± 0.28 b

a, b Data in the same row with different superscripts are significantly different at *p* ≤ 0.05.

**Table 2:** Effects of various concentrations of NR-PS on the expression of cell surface antigens on BMHC-imDCs.

Groups	Percentages of cells with a positive cell surface antigen reaction *	

	CD 80/86 **	MHC II **
Control	65.45 ± 0.97	34.87 ± 1.96
LPS (μg/mL)		
1	85.76 ± 3.06	73.20 ± 6.16
NR-PS (μg/mL)		
3.125	84.49 ± 10.86	45.06 ± 8.11
6.25	84.80 ± 7.94	44.88 ± 5.11
12.5	85.68 ± 18.66	49.18 ± 10.41
25	87.16 ± 8.49	52.01 ± 10.11
50	84.44 ± 6.35	49.27 ± 5.03
100	84.56 ± 7.62	50.27 ± 5.24

* Cell surface antigen expression was analyzed by flow cytometry and data in the same column with different superscripts are significantly different with *p* ≤ 0.05;

** Data are expressed as mean ± SD: *n* = 3.

**Table 3:** Comparison of the mean fluorescence intensity of dendritic cells across various concentrations of NR-PS.

Groups	Mean fluorescence intensity of dendritic cells *	

	CD 80/86 **	MHC II **
Control	24.60 ± 3.19	46.55 ± 4.97
LPS (μg/mL)		
1	49.00 ± 9.18	57.62 ± 1.87
NR-PS (μg/mL)		
3.125	47.23 ± 3.72	51.09 ± 4.73
6.25	42.73 ± 4.20	51.94 ± 2.89
12.5	45.69 ± 3.58	55.62 ± 8.21
25	48.48 ± 4.26	60.74 ± 10.06
50	38.03 ± 6.27	57.30 ± 4.76
100	43.70 ± 7.61	57.70 ± 2.68

* Cell surface antigen expression was analyzed by flow cytometry and data in the same column with different superscripts are significantly different with *p* ≤ 0.05;

** Data are expressed as mean ± SD: *n* = 3.

**Table 4:** Effect of various concentrations of NR-PS on the pinocytosis activity of BMHC-imDC.

Groups	Uptake of FITC-dextran *

	Mean fluorescence intensity **
Control 37 °C	261.67 ± 47.26
LPS (μg/mL)	
1	121.60 ± 31.05
NR-PS (μg/mL)	
3.125	227.87 ± 35.88
6.25	192.47 ± 52.47
12.5	176.50 ± 28.52
25	167.94 ± 60.59
50	199.41 ± 24.52
100	211.49 ± 33.65

* Pinocytosis activity was measured by the uptake of FITC-dextran (40,000 dalton; Sigma) and analyzed by FACScan flow cytometry. The data in the same column with different superscripts are significantly different at *p* ≤ 0.05;

** Data are expressed as mean ± SD: *n* = 3.

**Table 5:** Effect of various concentrations of NR-PS on IL-12, IL-10 and IFN-γ production by BMHC-im DCs.

Groups	Level of cytokine * (pg/mL)		

	IL-12 **	IL-10 **	IFN-γ **
Control	102.09 ± 10.16	30.75 ± 3.35	11.76 ± 0.11
NR-PS (μg/mL)			
3.125	158.59 ± 37.85	23.20 ± 8.47	15.40 ± 1.52
6.25	221.40 ± 11.90	22.48 ± 5.44	15.33 ± 2.62
12.5	220.65 ± 17.09	18.16 ± 4.17	15.96 ± 2.66
25	258.78 ± 25.26	15.37 ± 2.35	15.51 ± 1.66
50	254.62 ± 36.22	14.83 ± 3.80	15.01 ± 1.47
100	194.48 ± 27.61	21.22 ± 2.11	15.67 ± 1.24

* IL-12, IL10 and IFN-γ levels in the supernatants by ELISA. Data in the same row with different superscripts are significantly different at *p* ≤ 0.05;

** Data are expressed as mean ± SD: *n* = 3.

## Abbreviations

NR-PS: *Nymphaea rubra* Roxb. Polysaccharides
DCs: dendritic cells
BMHCs: bone marrow hematopoietic cells
BHMC-imDCs: BMHC-derived immature dendritic cells
DP: degree of polymerization
TCA: trichloroacetic acid
LPS: lipopolysaccharides
DB: degree of branching
